# Sensing spectrum sharing based massive MIMO radar for drone tracking and interception

**DOI:** 10.1371/journal.pone.0268834

**Published:** 2022-05-20

**Authors:** Milembolo Miantezila Junior, Bin Guo

**Affiliations:** School of Electronics and Information Engineering, Changchun University of Science and Technology, Changchun, Jilin, 130022, China; Sri Eshwar College of Engineering, INDIA

## Abstract

Radar sensors are becoming crucial for environmental perception in a world with the tremendous growth of unmanned aerial vehicles (UAVs) or drones. When public safety is a concern, the localization of drones are of great significance. However, a drone used for a wrong motive can cause a serious problem for the environment and public safety, given the fact that the dynamic movement of a drone’s emission signal and location tracking is different from existing positioning. This study proposes a safety zone characterized by the presence of N radars sensors with a goal to track and destabilized rogue drones attending to penetrate safety zones (stadium and school). Specifically, a new joint estimation based on a Gaussian filter has been introduced for spectrum sharing and detection awareness. The profit of this novel sensing method can be clearly seen when the two joint hidden states are taken into consideration. Therefore, the drone’s emission state is analyzed by estimating its movement jointly. Considering the drone’s unknown states and actual positioning, an algorithm is developed based on dynamic states space model. Where Bernoulli filter model is designed to estimate recursively the unknown stages of the drone and its changing location based on time. Meanwhile a power control acted from the radar to the targeted drones so that rogue drones are optimally tracked and destabilized over time. Furthermore, an expanding mechanism has been generated to accurately track the drone and enhance detection. A thoughtful result of the experimentation shows clearly that, even when the drone is moving, spectral detection can be performed accurately by chasing its positions. Its demonstrates at 90% of credibility that the original signal has a direct effect on the propagated signal. Therefore, the magnitude of the Doppler shift increases with frequency. And the clue of its positioning can be used for cognitive radio optimization.

## 1. Introduction

The utilization of drones has increased exponentially with rising technology in recent years. However, unmanned aerial vehicles (UAVs) or drones can become extremely dangerous for public safety and people’s privacy, when others applications and tools are added unto it such as urveillance, agriculture data analysis, movie making, mineral exploration without control and good monitoring [1]. Therefore, careful studies in UAVs and spectrum access are of greater importance. A deep analysis of anti-drone systems has been presented in [2,3], where a scenario of drones cooperating in order to track and destabilize rogue drones. The target (rogue drones) displays a stochastic dynamic movement and their trajectory overtime needs to be estimated from noisy sensor measurements. It also specified that the mobile agents show a limited sensing range, and that they can detect the presence of the rogue drones inside their sensing range with a probability of less than one. Consequently, due to the sensing limitation, it is well-noted that, in addition to the target measurements, the mobile agents receive false-alarm measurements as well. Recently, there has been a significant research-based on target detection and the use of radar micro-Doppler [4–11].

In [12–14] studies, an analysis based on TVDs (Time Velocity Diagrams) of small helicopters and multicopters, reveals that both are from simulations and measurements (X-band radars). The authors presented the properties of a single rotor and multiple rotors, with an even and odd number of blades, and with short and long integration time. Even though the system performs well, the on-ground and simulation tests are limited due to the lack of supplementary parameters such as the change of weather or environmental changes. In [15], the authors presented a Doppler spectrum access without time resolution. The Doppler spectrum is then used by a boosting classifier. The simulation has been executed at where the radar signal was generated from a moving helicopter. Its inefficiency lies on the fact that radar signal and target (drones) are moving at the same time and this will cause some detections problem. A useful ultra-wideband (UWB) Antenna for UAV applications has been proposed in [16,17], where antennas with a reflector are used to increase the gain at 2.4 GHz for UAV utilization and a monopole antenna that operates at 800 MHz was analyzed. In [18], a Pedestrian Dead Reckon (PDR) structure based on Inertial Navigation System (INS) sensor and UWB system was analyzed, where a modified zero-velocity detection algorithm and Kalman-type filter was developed to get the best angle by coupling zero-velocity information and single UWB. In [19], a mapping antenna array was presented with a circular polarization at the frequency range of 1.5Ghz to 1.65Ghz.A low-profile antenna structure was proposed in [20], where a Rogers Duroid 5880LZ material with dimensions of 29mm X 39mm was applied.

Experimental research based on multistatic passive radar with a single antenna for drone detection has been presented in [21], where the dominant direct-path signal (the strongest static clutter) in the reference channel was considered as an effective signal [22–26] and a scenario was proposed to utilize a compact single antenna receiver for the UAV detection. In this paper, we addressed a spectrum access for massive multi-input multi-output (MIMO) radar covering a safety zone for rogue drones intrusion. Here, MIMO radar and UAV (drone) share interferences on the same spectral zone. Our main goal here is to detect and track until the destabilization of the target (drones). The contribution of this work is summed up as follows:

1. The localization tracking of the drone with unknown emission states has been investigated and a new sensing technique has been proposed to estimate the localization of the drone and at the same time detect its spectral position. Specifically, in this new technique the tracking of the drone cannot be interrupted even though its emission states are suspended. Therefore, this technique came to break the traditional method which doesn’t consider the dynamic emission states of the drone.
2. Novel algorithm based on massive MIMO radar and drone spectrum sensing which rely on dynamic Bayesian filter approach. The particularity of this approach is that, the unknown emission state of the drone is analyzed as an additional hidden state that needs to be analyzed, instead of its changing locations. Considering the limited information available in spectrum access, we used received signal strength to estimate recursively the two hidden states. Meanwhile, this technique can also be extended in other scenario such as spectrum access between MIMO radar, 5G Communication system, and drone localization.
3. A Soft joint distribution algorithm has been developed, where the emission state of the drone and other associated state such as its unknown positions, are analyzed like Bernoulli random finite set (BRFS). We took the advantage of Bayesian assumption algorithm to estimate recursively drone’s existence state and its dynamic positioning,which most of the times in real live are difficult to analyze. To enhance the tracking scenario of the drone when its goes off, a horizon analysis was developed, which can be adjusted prior to uncertainty inference process. It is demonstrated by an extensive numerical experimentation that, the system is not only efficient but also in estimating the drone’s location and detection, uncertainty of reception can be measured, therefore, the system can constantly be optimized.

The rest of the paper is organized as follows: the sensing methodology, dynamical states, dynamical positioning, and statistical detection of the drone is presented in section 2. In Section 3, we presented numerical experiment and performance analyses. We discussed the performance of the sytem and Algorithm scenarios in section 4. Section 5 is the conclusion of the paper.

## 2. Materials and methods

### 2.1. System model

**Fig 1:**
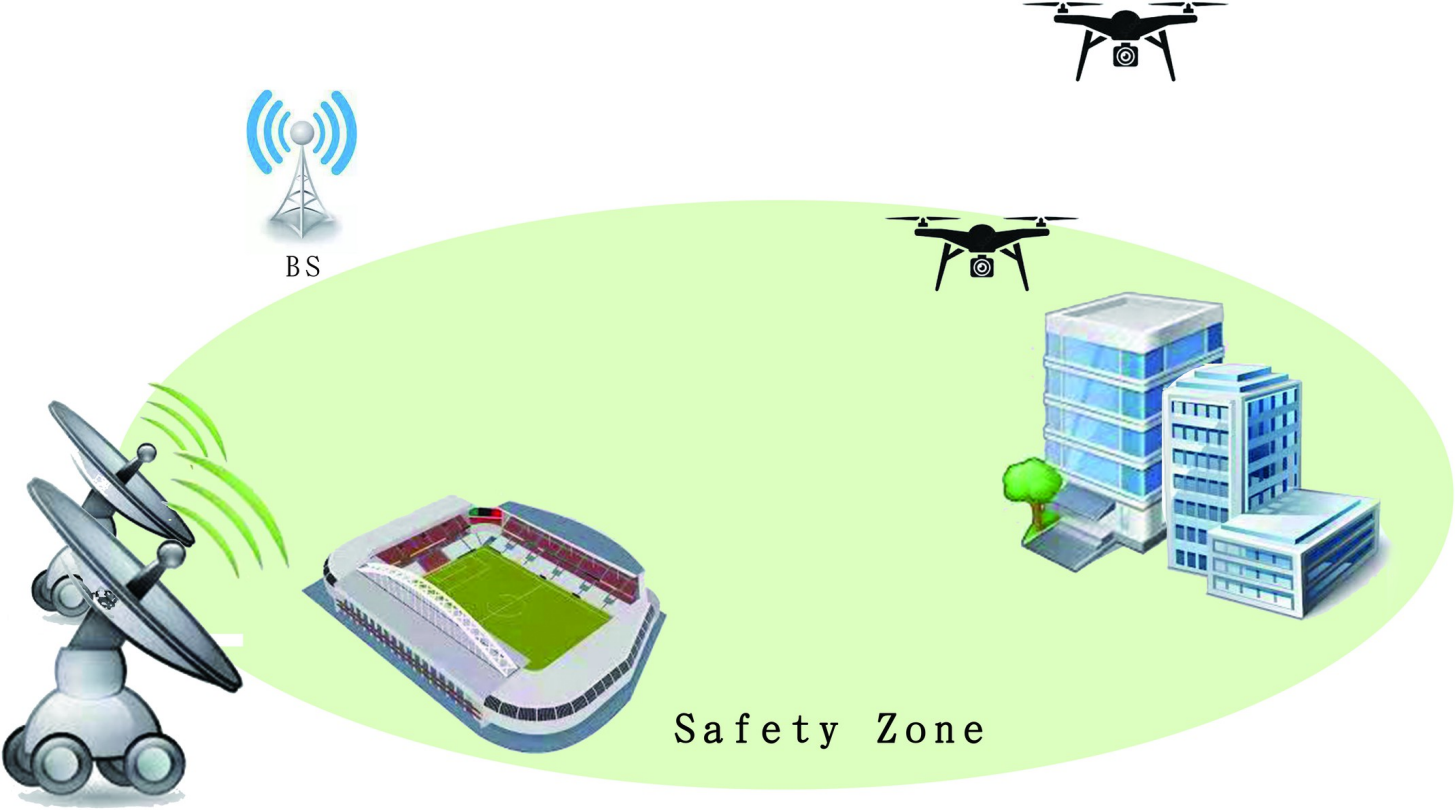
Spectrum sharing for massive MIMO radar and drones detection entering safety zone on brownian motion.

By considering simultaneous observation from spectrum sensing of MIMO radar and drone localization, we addressed a cooperative scenario as presented in Fig 1. Our drone system is moving as Brownian models continuous motion [27]. For a better analysis and representation, we denote *M*, a cooperative MIMO radar in cartesian coordinate, with position of each node noted by a_m_ = [*x*_*m*_, *y*_*m*_]^t^(m = 1,2, 3,…M).

We considered this information to be previously known by the data center. To perform spectrum sensing and drone localization at the same time, a two-step scenario scheme were selected. In the first step, the *m*th MIMO radar antenna will intercept the nearest wireless network at each time discrete t, and receive the information about the local observation *o*_*t*,*m*_. In the Following step, all MIMO radar node will send their observation data information to the data center for analysis. The Information will then be compiled and the observation will be extracted based on the observation vector *o*_*t*_ = ${\left[o_{t_{1}},o_{t_{2}},\ldots ,o_{t_{M}}\right]}^{\text{t}}$ and the emission state of the drone positions will be estimated $r_{t,}\left(x_{t}^{'},y_{t}^{'}\right)$.

#### 2.1.1. Sensing method

For easy analysis, the dynamic notation can be summarized as,

$$r_{t}=R\left(r_{t-1}\right)$$ (1)

*R*(.) is a dynamic function ℝ→ℝ which specifies stochastic progress of drone’s emission states *R*. By considering the fact that drone is an agent vulnerable to any movement and external influence while in the air, we define two transitional functions ℝ→ℝ,

$$v_{t}=V\left(v_{t-1},h_{1}\right)$$ (2)

$$\theta_{t}=\theta \left(\theta_{t-1},h_{2}\right)$$ (3)

These two random stochastics represent the behaviors of drone speed movement *v*_*t*_ and angular orientation *θ*_*t*_ while in the air, which are moved independently and randomly by noises *h*_1_ and *h*_2_ respectively. Drone still in the air is a dynamic agent, which means we will have to define its dynamic movement,

$$U_{t}=I\left(U_{t-1},v_{t},\theta_{t}\right)$$ (4)

Where *I*(.) is the transition function ℝ^2^ → ℝ^2^, specifies the dynamics drone’s movement with the vector location $U_{t}={\left[x_{t}^{'},y_{t}^{'}\right]}^{\text{t}}$,and the observation function,

$$o_{t}=O\left(U_{t},r_{t},w_{t}\left(n\right)\right)$$ (5)

*o*_*t*_ is the measurement equation with observation function *0*(.): ℝ^*M*^→ ℝ^1^,which describes the relationship between two hidden states *r*_*t*_, *u*_*t*_ and the measurement *o*_*t*,*m*_.

From here, three assumptions have been made to execute the sensing. First, a segment of a periodic sensing is performed, where the emission state of the drone is assumed to remain fixed. This means *r*_*t*_ will remain unchanged for one sensing period of *T*_*r*_,after that it will change.

Secondly, the static Gaussian filter was considered at this stage. The observation *o*_*t*,*m*_ is relative between the *m*th radar and the moving drone. The noise random estimation of the *n*th portion at discrete time *t* noted as *w*_*t*_(*n*) of Eq (5) is assumed to be independent identical distribution with zero mean additive white Gaussian noise, where variance is $\sigma_{w}^{2}$,which is also independent identical distribution of two hidden states.

Thirdly, we considered the drone as moving with positioning $U_{t}={\left[x_{t}^{'},y_{t}^{'}\right]}^{\text{t}}$ and its remain constant during a period of time *T*_*r*_.

#### 2.1.2. Drone’s dynamic states

After analysis, we find out that the progress of emission states of drone over time *T*, can be represented as a finite states engine and can be described as two states Markov process [28–32] *R* = {*R*_0_, *R*_1_}. If we consider the drone as active and moving with emission states *R*_1_ at time *t*, then the survival probability of an active drone can be written,

$${\text{P}}_{\text{r}}\Rightarrow Pr\left[r_{t+1}=1\;\backslash {\text{r}}_{\text{t}}=1\right]$$ (6)

$${\text{P}}_{\text{r}}\Rightarrow Pr\left[r_{t+1}=1\;\backslash {\text{r}}_{\text{t}}=1\right]=\lambda_{t}\Delta t+o\left(\Delta t\right),$$

Where *λ*_*t*_ is the survival rate. Dealing with a Markov process, the probability of transition will depend on only the current state. We can determine the probability of the drone remaining in its survival movement by adding all the probabilities of its ways of progress:

$${\text{P}}_{\text{r}}\left(r_{t+1}=1\right)=Pr\left[r_{t+1}=1\;\backslash {\text{r}}_{\text{t}}=1\right]P\left(r_{t}=1\right)$$

$$\begin{array}{l} \text{Pr}\left[r_{t+1}=1\backslash r_{t}=2\right]P\left(r_{t}=2\right)+\\ \ldots +\text{Pr}\left[r_{t+1}=1\backslash r_{t}=\text{r}\right]P\left(r_{t}=\text{r}\right) \end{array}$$ (7)

The computation of Eq (7) can easily lead us to matrix notation. Then the vector of each survival probability can be written as,

$$p\left(t\right)=\left[\begin{array}{c} P\left(r_{t}=1\right)\\ P\left(r_{t}=2\right)\\ \vdots \\ P(\left(r_{t}=\text{r}\right) \end{array}\right]$$ (8)

And it is transition matrix can be represented as,

$$A=\left[\begin{array}{c} P\left(1\backslash 1\right)\;P\left(1\backslash 2\right)\ldots P\left(1\backslash r\right)\\ P\left(2\backslash 1\right)\;P\left(2\backslash 2\right)\ldots P\left(2\backslash r\right)\\ \vdots \\ P\left(r\backslash 1\right)\;P\left(r\backslash 2\right)\ldots P\left(r\backslash r\right) \end{array}\right]$$ (9)

The drone will go into sleeping mode on states *R*_0_ with a probability 1 − P_r_ in the following time *t+1*. If the drone stays in sleeping mode *R*_0_, it will move again into *R*_1_ states with a birth probability,

$${\text{P}}_{\text{b}}\Rightarrow Pr\left[r_{t+1}=1\;\backslash {\text{r}}_{\text{t}}=0\right]$$ (10)

$${\text{P}}_{\text{b}}\Rightarrow Pr\left[r_{t+1}=1\;\backslash {\text{r}}_{\text{t}}=1\right]=\mu_{t}\Delta t+o\left(\Delta t\right),$$

Where *μ*_*t*_ is the birth rate and it may remain in states *R*_0_ in the next time with a probability 1 − P_b_. In the same way, we can determine the probability of the drone remaining in its birth probability by adding all the probabilities of its ways of maintenance:

$${\text{P}}_{\text{r}}\left(r_{t+1}=1\right)=Pr\left[r_{t+1}=1\backslash {\text{r}}_{\text{t}}=0\right]P\left(r_{t}=0\right)$$

$$\begin{array}{l} \text{Pr}\left[r_{t+1}=1\backslash r_{t}=1\right]P\left(r_{t}=1\right)+\\ \ldots +\text{Pr}\left[r_{t+1}=1\backslash r_{t}=\text{r}-1\right]P\left(r_{t}=\text{r}-1\right) \end{array}$$ (11)

Then the vector and transition matrix of each birth probability can be written respectively as,

$$p^{\prime }\left(t\right)=\left[\begin{array}{c} P\left(r_{t}=0\right)\\ P\left(r_{t}=1\right)\\ \vdots \\ P\left(r_{t}=\text{r}-1\right) \end{array}\right]$$ (12)

$$A^{\prime }=\left[\begin{array}{c} P\left(1\backslash 0\right)\;P\left(1\backslash 1\right)\ldots P\left(1\backslash r-1\right)\\ P\left(2\backslash 0\right)\;P\left(2\backslash 1\right)\ldots P\left(2\backslash r-1\right)\\ \vdots \\ P\left(r\backslash 0\right)\;P\left(r\backslash 1\right)\ldots P\left(r\backslash r-1\right) \end{array}\right]$$ (13)

It is worthy to note that in the above mentioned dynamic probability, the transitional matrix is specific with the drone devises. In other wireless devises the dynamic transition remain invariant for a longer period T [33].

#### 2.1.3. Drone’s dynamic positioning

Firstly, statistical action of the speed and orientation of the drones were studied, where it was realized that the drone is moving following a random walking process. As two random variables, the speed and orientation Eqs (2) and (3) at time t can be written as,

$$v_{t}=v_{t-1}+h_{1}\;h_{1}\sim N\left(0,\sigma_{v}^{2}\right)$$ (14)

$$\theta_{t}=\theta_{t-1}+h_{2}\;h_{2}\sim \varepsilon \left(0,\sigma_{\theta }^{2}\right)$$ (15)

Where $\sigma_{v}^{2}$ and $\sigma_{\theta }^{2}$ represent the variances of drone’s acceleration and direction, respectively. We consider that the two noises $N\left(0,\sigma_{v}^{2}\right)$ and $\varepsilon \left(0,\sigma_{\theta }^{2}\right)$ which are Gaussian, are following the path of random walking. By considering the above equations, based on speed and orientation of the drone, we can then represent the dynamic cartesian equations of its position by,

$$x_{t}=x_{t-1}+v_{t}cos\left(\theta_{t}\right)$$ (16)

$$y_{t}=y_{t-1}+v_{t}sin\left(\theta_{t}\right)$$ (17)

Where *x*_*t*_ and *y*_*t*_ represent the abscissa and ordinate of the cartesian axes position of the drone respectively.

#### 2.1.4. Statistical detection

In order to derive a decision rule and the detection analysis, which maximizes *Pr*[*r*_*t*+1_ = 1\r_t_ = 1]. Based on the observation set *o*_*t*,*m*_, given this realization, the conditional probability of correct detection can be written as,

$$Pr\left[r_{t+1}=1\;\backslash {\text{r}}_{\text{t}}=o_{t,m}\right]$$

and the observed signal in practice can be represented as,

ot,m=∑n=1NρtEratndt,m−α2+wtn2 (18)

Where *o*_*t*,*m*_ is the received signal strength of the *m*th radar, *d*_*t*,*m*_ is the distance that separate the *m*th radar and the stirring drone at time *t*, α is the path loss fading which supposed to be greater than 2. *ρ*_*t*_ represent the received gain of the *m*th radar which is from radar processing devises. *N = Tƒ* is the samples size and ƒ is the sampling frequency. *a*_*t*_(*n*) is the progression of drone’s message indications, where *n* = 1,2, ‥‥, *N*. For easy analysis, binary phase shift keying (BPSK) has been considered, where *a*_*t*_(*n*) = {+1, -1}, with *E*_*r*_ the emission power. For the absence of drone, the received signal strength is simply,

$$o_{t,m}=\sum_{\text{n}=1}^{\text{N}}w_{t}{}^{2}\left(n\right)$$ (19)

With a moving drone, the observation also may continue to be uncertain. Therefor an Euclidean distance between the targeted agent, e.g. drone and the radar are for a greater importance.


$$d_{t,m}\Rightarrow {\left|\left|U_{t}-a_{m}\right|\right|}_{2}=\sqrt{{\left(x_{t}^{'}-x_{m}\right)}^{2}+{\left(y_{t}^{'}-y_{m}\right)}^{2}}$$ (20)

By considering the distance *d*_*t*,*m*_ and drone’s emission states r_t_, the component likelihood density can be written as *p*(*o*_*t*,*m*_\*d*_*t*,*m*_,r_t_). As the *N* has to be very huge (such as, *N* ≥ 100), we can estimate the likelihood functions by applying Gaussian densities of i.i.d noise. The central limit theorem (CLT) will give the following approximations,

$$p\left(o_{t,m}\backslash U_{t},{\text{r}}_{\text{t}}\right)\left\{\begin{array}{c} \overset{M}{\underset{m=1}{\prod }}p\left(o_{t,m}\backslash U_{t},{\text{r}}_{\text{t}}=1\right)\sim {\text{H}}_{1}\\ \overset{M}{\underset{m=1}{\prod }}p\left(o_{t,m}\backslash U_{t},{\text{r}}_{\text{t}}=0\right)\sim {\text{H}}_{0} \end{array}\right.$$ (21)

Consequently, all observations from the data center can also be seen as Gaussian distribution with mean and variance respectively,

$$\varphi \left(o_{t}\backslash U_{t},r_{t}\right)=\overset{M}{\underset{m=1}{\sum }}\varphi \left(o_{t,m}\backslash U_{t},r_{t}\right)$$

$$\vartheta \left(o_{t}\backslash U_{t},r_{t}\right)=\overset{\text{M}}{\underset{\text{m}=1}{\sum }}\vartheta \left(o_{t,m}\backslash U_{t},r_{t}\right)$$

#### 2.1.5. Drone’s states prediction

As known, in the Bayesian approach [32,34], we analyse the unknown quantity, as a random variable. We recursively estimate the conditional posterior distribution.

*P*_*t*-1_(*r*_*t*-1_\*o*_1,*t*-1_) at time *t-1*. In our case the trajectory of the drone‘s emission states at *t*th discrete time is define by r = {*r*_0_, *r*_1_, …, *r*_*t*_}. Bayesian method is an effective mechanism to analyze and estimates hidden states. The prediction and updates of the posterior distribution of the hidden states *r*_*t*_ can be computed based on Bayes filter,

$$P_{t\backslash t-1}\left(r_{t-1}\backslash o_{1:t-1}\right)=\int p_{t\backslash t-1}\left(r_{t-1}\backslash r_{1:t-1}\right)p_{t-1\backslash t-1}\left(r_{t-1}\backslash o_{1:t-1}\right)dr_{t-1}$$ (22)

$$P_{t\backslash t}\left(r_{t}\backslash o_{1:t}\right)=\frac{p_{t}\left(o_{t}\backslash r_{t}\right)p_{t\backslash t-1}\left(r_{t}\backslash o_{1:t-1}\right)}{\int p_{t}\left(o_{t}\backslash r_{t}\right)p_{t\backslash t-1}\left(r_{t}\backslash o_{1:t-1}\right)dr_{t}}$$ (23)

Where Eqs (22) and (23) represent the prediction and update respectively, and function *P*_*t\t*-1_(*r*_*t*-1_\*o*_1:*t*-1_) and *P*_*t\t*_(*r*_*t*_\*o*_1:*t*_) represent the transitional density and likelihood function respectively. With the above assumption, the joint density can be estimated recursively. The ordinary estimation process for the sensing may become weak or raise concerns of imperfection due to drone’s constant changing position. It can be noticed that, the dynamic distance from Eq (20) may disappear completely by observing from the data center, when a drone goes off (i.e., *H*_0_ *or r*_*t*_ = *r*_0_). In analysing a Bayesian inference for an unknown position, the related likelihood involving the drone and radar distances may become unavailable, making the tracking of the drone’s dynamic position difficult to analyze. Another important aspect is that, without a clear drone’s positioning, the estimation of the drone states will be inaccurate. This is because of the imprecise result of the reception, especially for Energy Detection (ED) sensing method.

### 2.2. Random finite state

A Random Finite State (RFS) is a random variable that takes values as unordered finites sets [35]. The Effect of drone’s signal appearing or disappearing can possibly be treated as another aspect of random states [36–38]. In this present study, for a deeper analyses for dynamic behaviors of the drone, the two hidden states are studied like one combined random process called random finite state, represented as Φ [39].

The cardinality of a RFS Φ (i.e number of elements) is random and analyzed according to a discrete distribution *ρ*(*g*) = *P*{|*Φ*| = *g*}, where *g* ∈ ℕ_0_ and *g* = |*Φ*| is the cardinality of RFS Φ. A RFS Φ is characterized by its cardinality and a group of symmetric joint distribution [35,40] *ρ*(*Φ*_1_, …*Φ*_*g*_), *Φ*_1_, …*Φ*_*g*_ ∈ ℝ^*g*^.

According to the current drone sensing, |*Φ*_t_| ∈ {0.1} which means a binary threshold *γ*_*t*_ need to be taken into consideration, which stand for *γ*_*t*_ = 1 (i.e.,*H*_1_) when drone emitted a signal at time *t*, otherwise *γ*_*t*_ = 0 (i.e.,*H*_0_). Consequently, it was noticed that the random variable *γ*_*t*_ and the cardinality distribution *ρ*(*g*) are Bernoulli RFS. The Bernoulli RFS can either be empty (with probability 1 − *q*) or have one element (with probability *q*). According to Mahler’s theorem [39,40], the probability density function (PDF) of the finite set statistics (FISST) for such Bernoulli RFS can be described as,

$$\rho \left(g\right)=\left\{\begin{array}{c} 1-q\;\text{if}\sim \;\Phi_{t}=\emptyset \;or\;\gamma_{t}=0\\ q\;\;\text{if}\sim \;\Phi_{t}=\left\{U_{t}\right\}\;or\;\gamma_{t}=1 \end{array}\right.$$ (24)

The probability density function (PDF) *p*(*Φ*_*t*_) can farther be developed as a normal random variable [40] as,

$$p\left(\Phi_{t}=\left\{\Phi_{1},\ldots ,\Phi_{g}\right\}\right)=g!\rho \left(g\right)p\left(\Phi_{1},\ldots ,\Phi_{g}\right)$$

Applying the set integral, we will have,

$$\int p\left(\Phi_{t}\right)\delta \Phi =p\left(\emptyset \right)+\overset{\infty }{\underset{t+1}{\sum }}\frac{1}{t!}\int p\left(\Phi_{1},\ldots ,\Phi_{g}\right)d\Phi_{1},\ldots ,d\Phi_{g}\equiv 1$$ (25)

It is clear to see that *p*(*Φ*_*t*_) integrate to one as it is should be for a PDF.

Thus said, it can further be seen that, the presence of a moving drone during the sensing can be represented as |*Φ*_*t*_| = 1, which correspond to the dynamic position *U*_*t*_.

Based on cardinality distribution *ρ*(*g*) and states distribution PDF *p*(*U*_*t*_), the FISST can be redefined as,

$$\rho \left(\Phi_{t}\right)=\left\{\begin{array}{c} 1-q\;\text{if}\sim \;\Phi_{t}=\emptyset \;or\;\gamma_{t}=0\\ q\;.\;p\left(\zeta \right)\;if\sim \;\Phi_{t}=\left\{U_{t}\right\}\;or\;\gamma_{t}=1 \end{array}\right.$$ (26)

For some cases where the cardinality *g* is greater than 1, then *p*(*Φ*_*t*_) = 0.

### 2.3. Dynamic transition agent

According to the actual system, the dynamic transitional model of the Bernoulli RSF *Φ*_*t*_ shall also follow Markov process. Thus said, the Eqs (8) and (12) can then be represented as,

$$p_{t\backslash t-1}\left(\Phi_{t}\backslash \left\{U_{t}\right\}\right)=\left\{\begin{array}{c} 1-p\left(t\right)\;\text{if}\sim \;\Phi_{t}=\emptyset \\ p\left(t\right)\pi_{t\backslash t-1}\left(U_{t}\backslash U_{t-1}\right)\;\text{if}\sim \;\Phi_{t}=\left\{\gamma_{t}\right\} \end{array}\right.$$ (27)

And

$$p_{t\backslash t-1}\left(\Phi_{t}\backslash \emptyset \right)=\left\{\begin{array}{c} 1-p^{\prime }\left(t\right)\;if\sim \;\Phi_{t}=\emptyset \\ p^{\prime }\left(t\right)b_{t\backslash t-1}U_{t}\;if\sim \;\Phi_{t}=\left\{U_{t}\right\} \end{array}\right.$$ (28)

Where *b*_*t*\*t*−1_ represent the birth and initial density when the drone is re-detected or re-emitting its signal. And *π*_*t*\*t*-1_ (*U*_*t*_\*U*_*t*-1_) is the dynamic survival transitional density of the drone’s location, which can be represented by [41]:

$$\pi_{t\backslash t-1}\left(U_{t}\backslash U_{t-1}\right)=\frac{1}{\sqrt{2\pi \sigma_{v}}}\exp\begin{array}{c} \left\{\frac{{\left({\left|\left|U_{t}-U_{t-1}\right|\right|}_{2}-v_{t-1}\right)}^{2}}{2\sigma_{v}^{2}}\right\}\\ \times \frac{1}{2\sigma_{\theta }^{2}}exp\left[-\frac{\left|\tan^{-1}\left(\frac{y_{t}-y_{t-1}}{x_{t}-x_{t-1}}\right)-\theta_{t-1}\right|}{\sigma_{\theta }^{2}}\right] \end{array}$$ (29)

Where tan^-1^(.) is the angular vector movement.

#### 2.3.1. Path loss

Current studies based on radio communications affected by large scale free space propagation model has proposed several path loss method [21,42]. In this current work, a single carrier frequency of 3.55Ghz has been adopted. Where more focus has been put on distance dependency. The Close-in free space reference (CI) path loss models can be expressed as [42],

$$PL_{CI}\left(d,f\right)=PL_{FS,ref}\left(f\right)+10n_{CI}\log_{10}\left(d\right)+\xi_{\sigma ,CI}$$ (30)

Where 10*n*_*CI*_ log_10_(*d*) is the logarithmic distance dependency behavior with *n*_*CI*_ path loss exponential (PLE). *ξ*_*σ*,*CI*_ represent the shadow fading in decibel and follows Gaussian distribution with zero mean with standard deviation σ. *PL*_*FS*,*ref*_ (*f*) represent the carrier frequency and it is calculated by applying Friis’s law for free space propagation:

$$PL_{FS,ref}\left(f\right)=20\log_{10}\left(\frac{4\pi f}{c}\right)$$ (31)

Where *c* is the speed of the light. And the doppler angular frequency,

$$f_{D}=\frac{2\nu_{r}f}{c}$$ (32)

Where *v*_*r*_ is the radial speed of the target.

### 2.4. Bernoulli filtering and control

This is very similar with Bayesian prediction and update, where the two posterior densities *p*_*t*\*t*_ (*Φ*_*t*_\*o*_1:*t*_) and *f*_*t*\*t*_ (*Φ*_*t*_) will be propagated recursively. On the prediction stage, the first prediction densities of the two terms *q*_*t*\*t*-1_ and *f*_*t*/*t*-1_(*U*_*t*_) can be derived as follows,

$$p_{t\backslash t-1}\left(\Phi_{t}\backslash o_{1:t-1}\right)=\int P_{t\backslash t-1}\left(\Phi_{t}\backslash \Phi_{t-1}\right)p_{t-1\backslash t-1}\left(\Phi_{t-1}\backslash o_{1:t-1}\right)\delta \Phi_{t-1}=P_{t\backslash t-1}\left(\Phi_{t}\backslash \emptyset \right)p_{t-1\backslash t-1}\left(\emptyset \backslash o_{1:t-1}\right)+\int P_{t\backslash t-1}\left(\Phi_{t}\backslash U_{t-1}\right)p_{t-1\backslash t-1}\left(U_{t-1}\backslash o_{1:t-1}\right)dU_{t-1}$$ (33)

Now we are solving *Φ*_*t*_ = ∅ (when the drone is off) with *p*_*t*\*t*-1_ (∅ \*o*_1:*t*-1_) = 1-*q*_*t*\*t*-1_ and *Φ*_*t*_ = {*U*_*t*_} (when the drone went on) with *p*_*t*\*t*-1_ (*U*_*t*_\o_1:*t*-1_) = *q*_*t*\*t*-1_ *f*_*t*\*t*-1_ (*U*_*t*_). And since the predicted FISST PDF is in the form of Eq (26), we will have:

$$q_{t\backslash t-1}=1-\left[\left(1-p_{b}\right)\left(1-q_{t-1\backslash t-1}\right)+\left(1-p_{s}\right)q_{t-1\backslash t-1}\right]$$

$$=p_{b}\left(1-q_{t-1\backslash t-1}\right)+p_{s}q_{t-1\backslash t-1}$$ (34)

Similarly, when the drone went on,

$$\begin{array}{l} f_{t\backslash t-1}\left(U_{t}\right)=\frac{p_{b}\left(1-q_{t-1\backslash t-1}\right)b_{t\backslash t-1}\left(U_{t}\right)}{q_{t\backslash t-1}}+\\ \frac{p_{s}q_{t-1\backslash t-1}\int \pi_{t-1\backslash t-1}\left(U_{t}\backslash U_{t-1}\right)f_{t-1\backslash t-1}\left(U_{t-1}\right)dU_{t-1}}{q_{t\backslash t-1}} \end{array}$$ (35)

It is worthy to note from the above two equations the predicted density (*q*_*t*-1\*t*-1_) and spatial density (*f*_*t*\*t*-1_ (*U*_*t*_) may involve two important elements, the birth element of a new drone appearing and a survival element of an already existing drone. The first birth is defined as the disappearance of the drone (e.g., *p*_*b*_); and the second one which is the survival is define by a continuing appearance of the drone (*p*_*s*_). The above two equations fully specify the step of Bernoulli filter.

## 3. Numerical results

**Fig 2:**
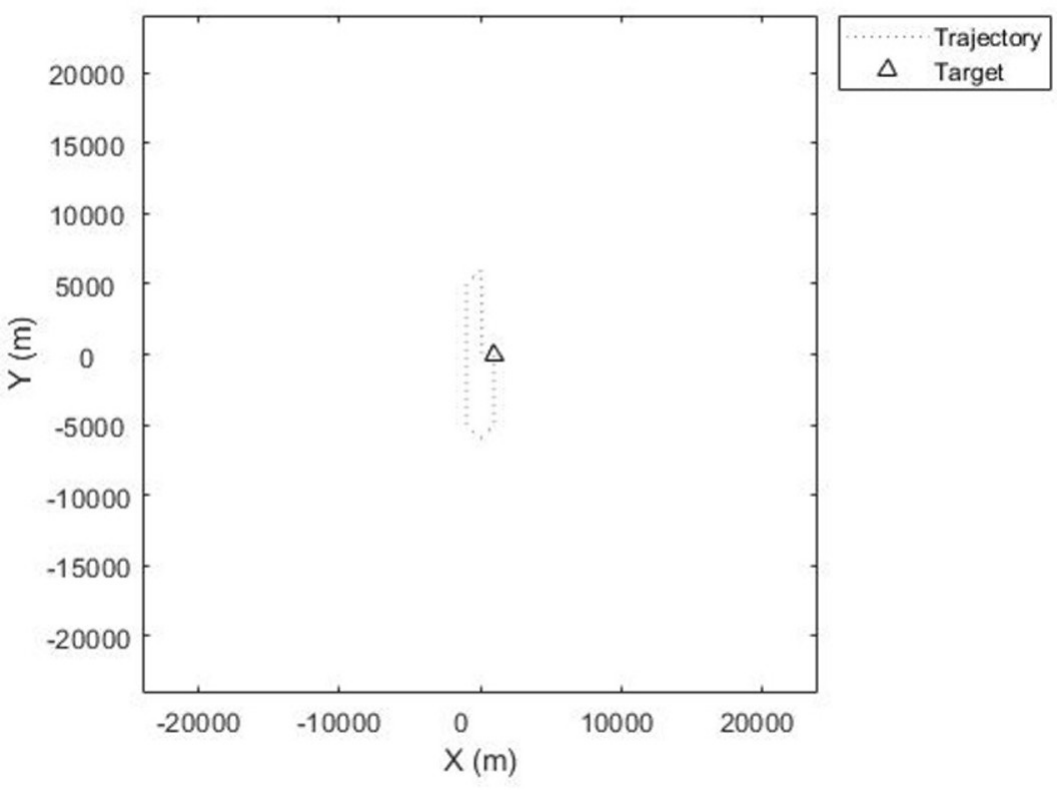
Target drone racetrack path with straight legs of 20 km and a turn radius of 2 km.

The results presented in this section are generated from Matlab Simulation and Simulink. These are more suitable for Dynamic and complex analysis because more parameters can be added. A dynamic radar detection of a targeted element (Drone), can just penetrate the zone of detection with its trajectory as shown in 2-D grid Fig 2. The first step was to generate a radar detection of a moving drone with straight legs of 20km and a turn radius of 2km. The altitude of the trajectory is 1km, which is defined as –1km by default North-East-Down coordination structure used in this scenario. The radar is mounted on a tower of 5m length, 5m width, and 30m of height. It is defined as spectrum origin [0,0,0]. A summary of notations presented in this paper can be found in Table 1.

**Table 1:** Massive MIMO radar parameters for test environment.

Parameters	Values
Radar & Drone Communication RF Band Radar Antenna Tx/Rx Iterations	3550–3650 MHz 4/2 1000
Carrier Frequency Update rate Turn Radius Radial Velocity Speed of Light	3.55 GHZ 5.5 Hz 2 Km 1000 m/s 3x10^8^ m/s
Target point Spectral efficiency(bits/sec/Hz) Sample Time Gravity	20 Km 1 0.02 9.8
Doppler angular frequency	4*v*_*r*_*f*/*c*
Path loss	*PL*_*CI*_(*d*, *f*)

**Fig 3:**
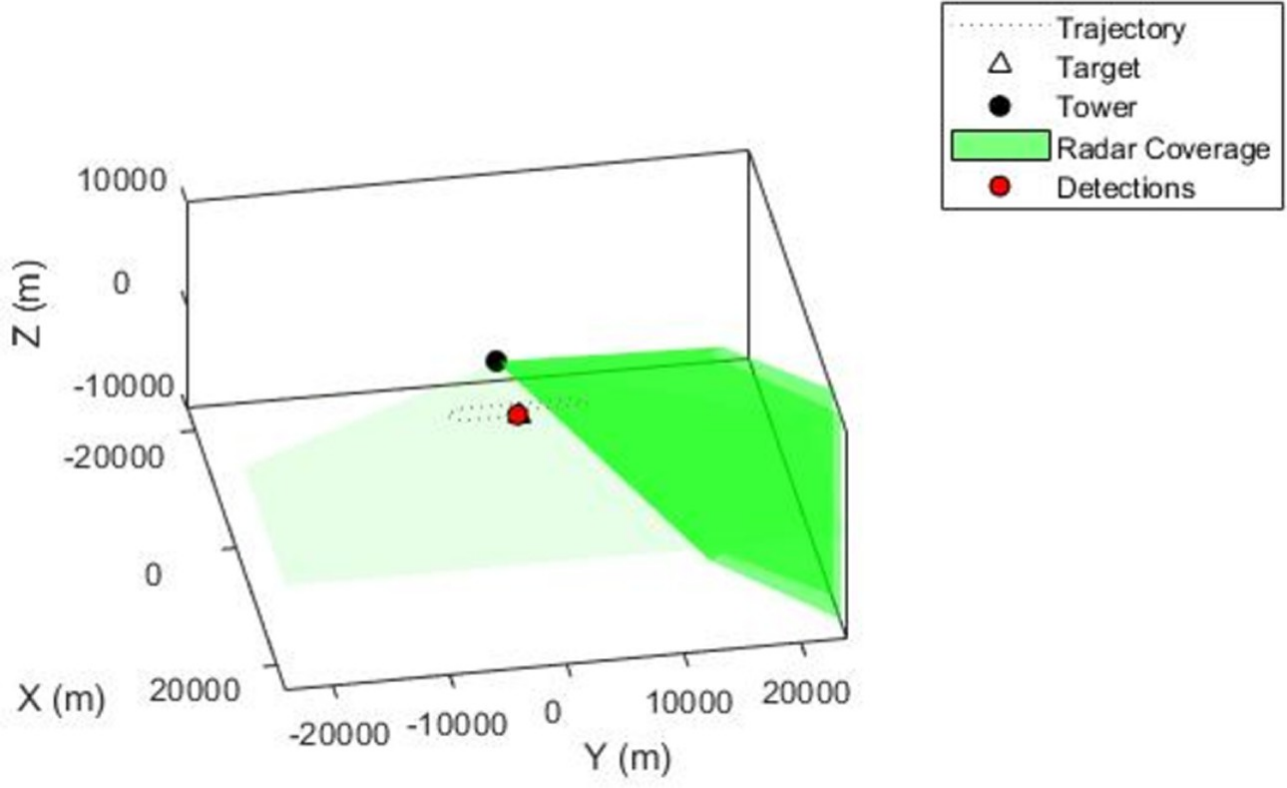
Plot of radar coverage mounted on 30m tower and signal detection of a moving drone.

A monostatic scanning radar sensor has been executed with the step size of update rate 5.5Hz in scene of 0.4 sec. Mounting location [0 0–15], field of view [4 45] and mechanical azimuth [–60–60]. The radar coverage zone with its scanning angle can be seen in Fig 3.

**Fig 4:**
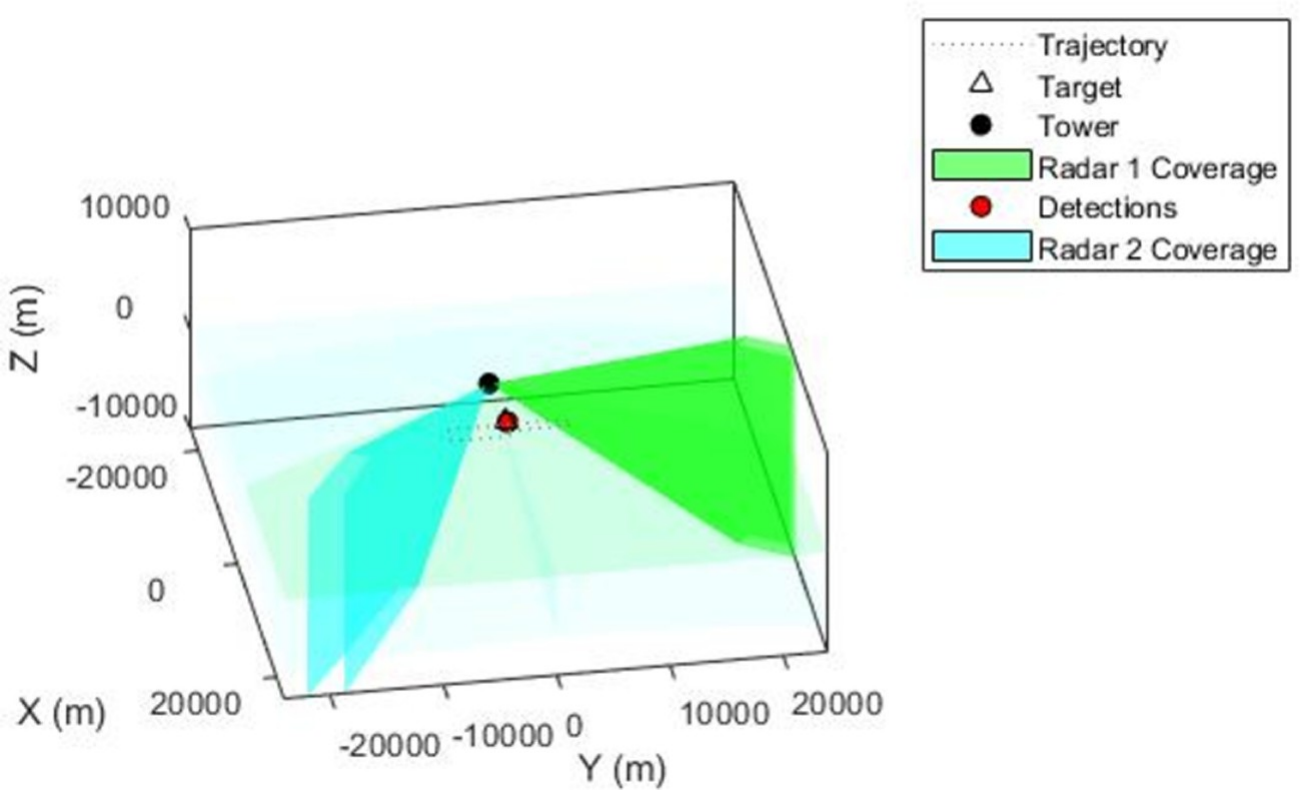
Second radar mounted on the tower. With it scanning detection angle and field of view of (4,45).

Secondly, another radar sensor has been added to the tower to amplify the detection in case of a huge intrusion of rogue drones in the protected area. It is added with an update rate 5.5Hz. And its performance is very high, as seen from its bleu scanning angle in Fig 4.

**Fig 5:**
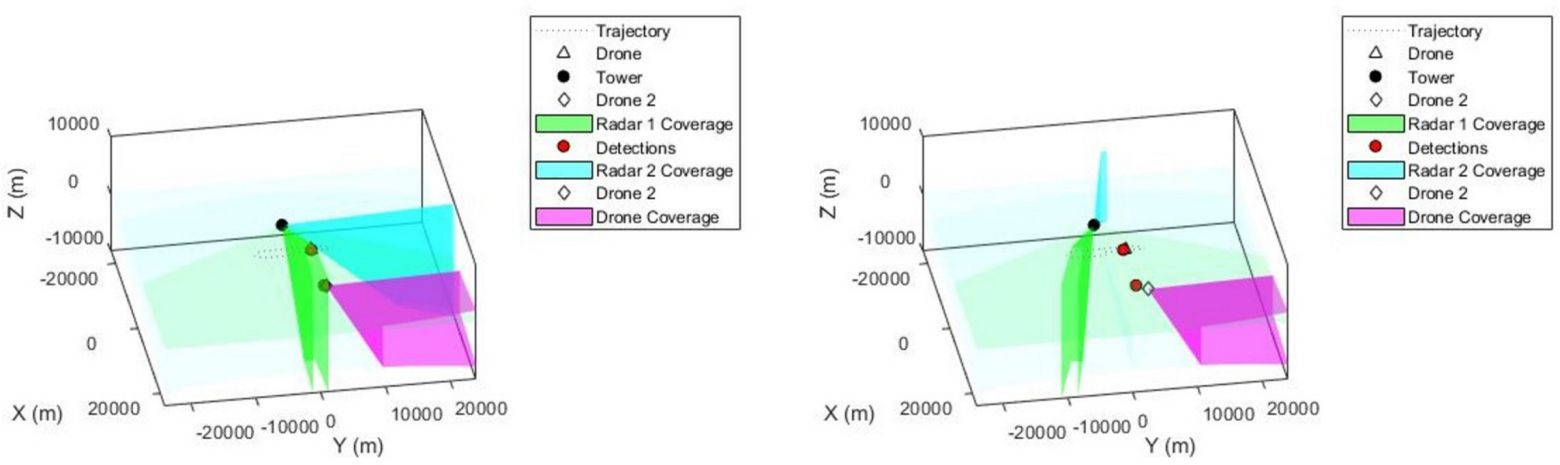
(**a**) Intrusion of second drone into the safety zone. Flying from southwest to northeast at a height of 1.5 km with a time of arrival [0 80]; (**b**) The second drone remain trackable. As its moves, its detection moves as well.

**Fig 6:**
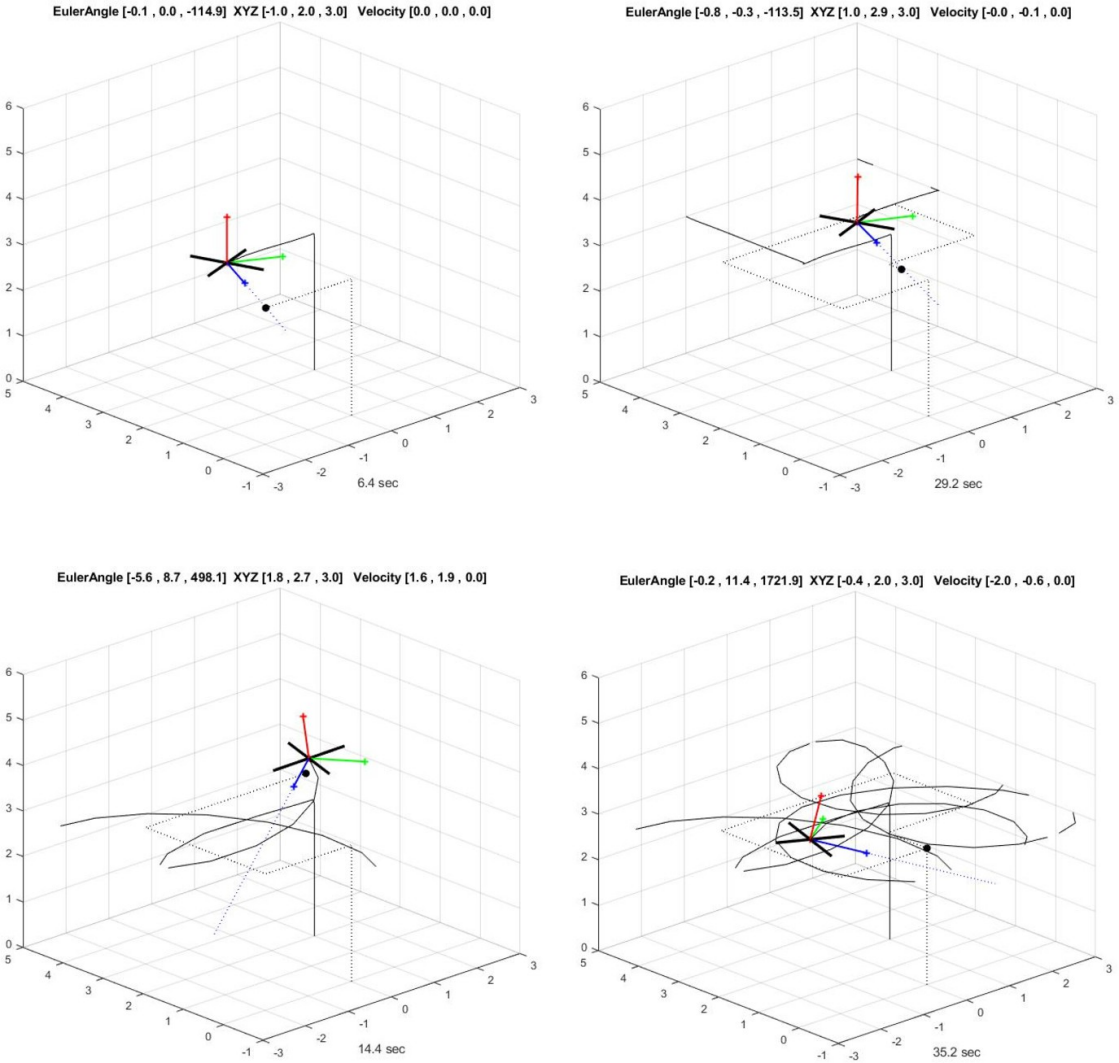
(**a**) Drone controlled followed square motion; (**b**) Drone controlled followed near radar on square motion; **(c)** Drone losing control as it received signal power of over 2.5 MHZ; **(d)** A drone that completely loses control after receiving a significant jamming power.

**Fig 7:**
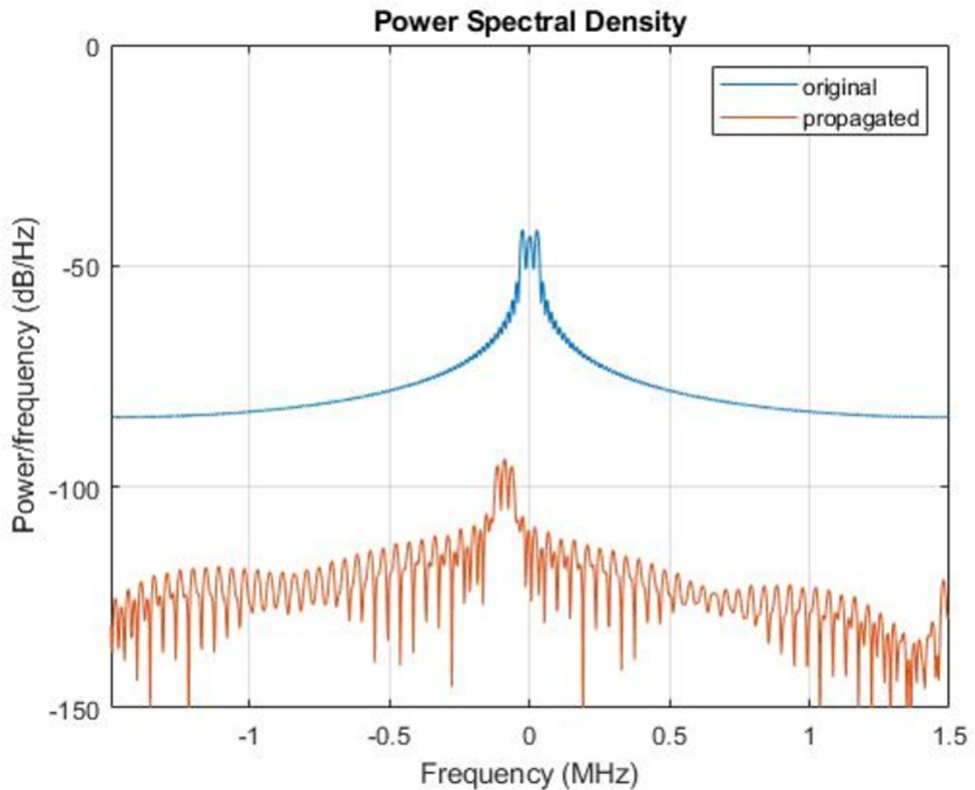
Plot of Wideband propagation signal for the spectrum of original signal and the Doppler-shifted signal with central frequency, 3Ghz.

**Fig 8:**
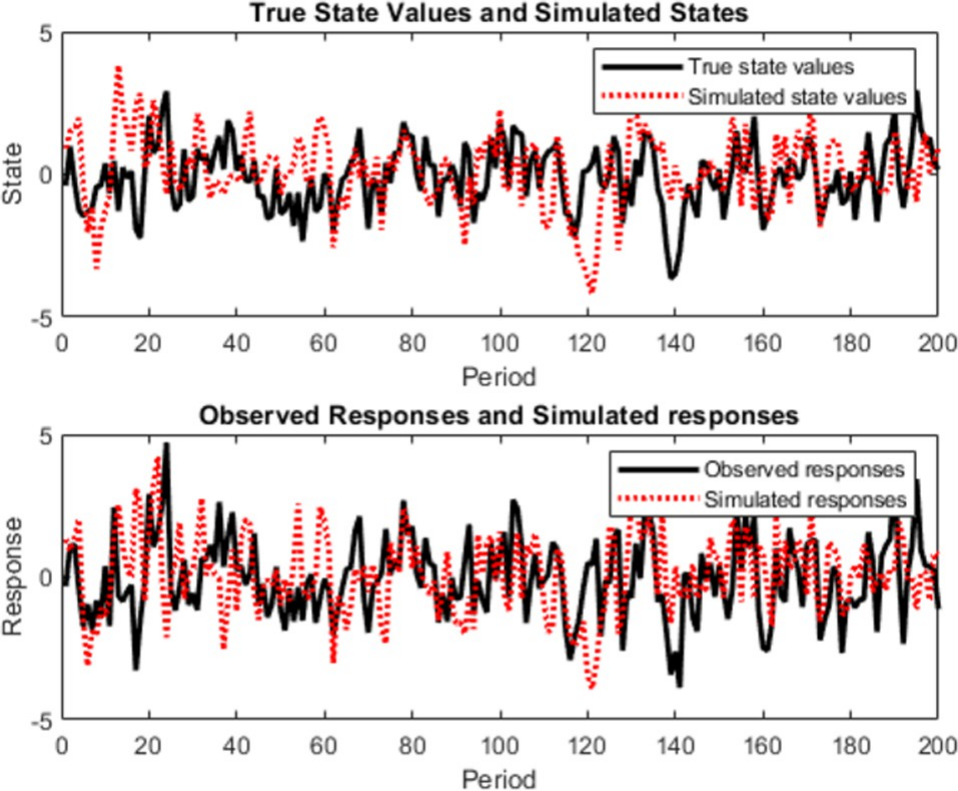
Plot of true state values and simulated states alongside with observed responses and simulated responses at a series of 200 observations.

In the third approach, there was an intrusion of a second drone in the safety zone, and this was quickly detected by the two radars as observed in Fig 5. The second drone flew from southwest to northeast at a height of 1.5km with a time of arrival [0 80]. All the reporting frames of the radars were sent back to the data center through inertial navigation system (INS). We noticed that, the second drone was equipped with a sensor which is able to inject anything to the safety zone. This leads to the next step, which is the destabilization of the drone or making it to turn back. A Proportional-Integral-Derivative (PID) control was applied for this scenario with parameters *R*(.),*V*(.),*θ*(.),*U*(.),*O*(.). The Euler initial position is defined by (0,0,0), with gravity (0,0, -9.8). As shown in Fig 6, the drone can be controlled following the radar position on a square model, Fig 6A and 6D and by doing so, a significant signal power of approximately 5 MHZ can be straight pointed to the target. However, the drone can progressively start losing its control as seen in Fig 6C and 6D. Fig 7 demonstrates the signal wideband propagation in a free space environment. The center frequency is 3 GHz and the frequencies of the three tones are 750 kHz, 1 GHz, and 1.5 GHz, respectively. The system model applies range-dependent time delay, gain adjustment, and phase shift to the input signal. Additionally, the model estimates the Doppler shift when the drone is moving. The free-space environment is a boundary-free medium with a speed of signal propagation independent of position and direction. The signal is propagated along a straight line from the source to its destination. Therefore, the model shows the two-way propagation of the signal from the radar to the targets. For this wideband signal, it was observed that the magnitude of the Doppler shift increased with frequency. In case of narrowband signals, the Doppler shift is assumed to remain constant over the band. Lasty, the system model performance has been tested as a series of states spaces model, to validate the model with real world. Based on time invariant, the simulate states and observations were estimated. As shown in Fig 8, the true state and simulated states of the target were compared to the observed responses and simulated responses from the radar at a series of 200 observations. It was shown that, the true states values of the target aligned to 90% with the observed values.

## 4. Discussion

The development of wireless and control system has accelerated the use of unmanned aerial vehicles (UAVs) or drones. However, public safety as well as privacy has become a general concern. In this study, we analyzed spectrum access for massive MIMO radar covering a safety zone for rogue drones’ intrusion, by considering numerous numbers of drones. Here, the MIMO radar and UAV (drone) shared interferences on the same spectral band zone. We present here a novel technique in detecting and tracking the targeted drones until their destabilization. This was made possible with a deep analyses of drone’s localization tracking, where the emission states of the drone is estimated. This is followed by dynamic Bayesian filter approach algorithm, based on massive MIMO radar and drone spectrum sensing. The particularity of this new approach is that, the unknown emission state of the drone is analysed like another hidden state that needs to be estimated, instead of the changing locations. Considering the limited information available in spectrum access, we used received signal strength to estimate recursively the two hidden states. Meanwhile, this technique is also very useful in scenarios of spectrum access between MIMO radar, 5G Communication base station system, and drone localization. A joint distribution algorithm based Bernoulli Random Finite Set (BRFS) has been developed, where the emission state of the drone and the associate state such as its unknown locations was analysed. We estimated recursively drone’s existence state and its dynamic positioning, in which most times it’s difficult to be analysed in reality in real live. This will enhance the tracking scenario of the drone when it appear and disappear from the scene. Corresponding to Algorithm summary flow, there were two main parts involved in this work:

1. By using Observation tracking trajectory *O*_1*t*_, and relying on recursive Bayesian Filter prediction and update, we estimated the posterior density *f*_*t*\*t*-1_(*U*_*t*_),
2. Tracking of detection uncertainty with length *r*_*t*-1_ = *R*_0_ as J, where 0 < *j* < *J* and the next step of state is define as *r*_*t*-*J*-1_ = *R*_1_. Therefore, drone goes collapsing in stage $\prod_{m=1}^{M}p\left(o_{t,m}\backslash U_{t},{\text{r}}_{\text{t}}=0\right)\sim {\text{H}}_{0}$ at time *t*—*J*, and its prediction estimation is subsequently t–J + m,(where m = 1,2,M). If the drone continues its move in stage $\prod_{m=1}^{M}p\left(o_{t,m}\backslash U_{t},{\text{r}}_{\text{t}}=1\right)\sim {\text{H}}_{1}$ at time t, a complete likelihood of the birth density is estimated,

$$b_{t\backslash t-1}\left(X_{t\backslash t-1}\right)\approx f_{\text{v}}\left(v_{t}\backslash J\right).f_{\theta }\left(\theta_{t}\backslash J\right)$$ (36)

### Algorithm Scenario

Iterate

Observation Information Collection,

*o*_*t*,*m*_ = $\sum_{n=1}^{N}w_{t}{}^{2}\left(n\right)$, Eq (19)

Estimate Dynamic transitional model RSF Φ_t_

p_t\t-1_ (Φ_t_\{U_t_}) Eq (27) and p_t\t-1_ (Φ_t_\∅) Eq (28)

Confirm Drone’s survival density: *π*_*t*\*t*-1_ (*U*_*t*_\*U*_*t*-1_}

Prepare Bernoulli Filters for prediction

*p*_*t*\*t*-1_ (*Φ*_*t*_\*o*_1__:__*t*-1_) Eq (33)

For if i = 1: M

 Compute recursive Bayesian Filter prediction

  *q*_*t*\*t*-1_ Eq (34)

   Update: f_*t*\*t*-1_(*U*_*t*_) Eq (35)

    Forward Eqs (34) and (35) to track

  detection uncertainty with length *r*_*t*-1_ = *R*_0_,

  where 0 < *j* < *J* and next step *r*_*t*-*J*-1_ = *R*_1_

End for

Learn Drone’s collapsing stage,

$\prod_{m=1}^{M}p\left(o_{t,m}\backslash U_{t},{\text{r}}_{\text{t}}=0\right)\sim {\text{H}}_{0}$

$i_{min}=\text{arg}\;\underset{1\leq \text{i}\leq \text{M}}{\text{max}}\begin{array}{c} \prod_{m=1}^{M}p\left(o_{t,m}\backslash U_{t},{\text{r}}_{\text{t}}=0\right)\sim {\text{H}}_{0} \end{array}$

If not, estimate the survival stage

$\overset{M}{\underset{m=1}{\prod }}p\left(o_{t,m}\backslash U_{t},{\text{r}}_{\text{t}}=1\right)\sim {\text{H}}_{1}$

By completing the birth density likelihood,

$b_{t\backslash t-1}\left(X_{t\backslash t-1}\right)\approx f_{\text{v}}\left(v_{t}\backslash J\right).f_{\theta }\left(\theta_{t}\backslash J\right)$ Eq (36)

End

It is demonstrated by an extensive numerical experimentation that the system is not only efficient but also potentially accurate in estimating the drone’s location and detection. The uncertainty of reception can be measured, therefore, the system can constantly be optimized.

## 5. Conclusions

As unmanned aerial vehicles are been used in our daily lives, a greater concent are been placed on human environment, privacy and public lives.

In this paper, a new approach base on spectrum sensing to realize drone’s tracking, detection, and destabilization for a safety zone and Cognitive Radio Applications has been proposed. A System model has been established to thoroughly characterize the dynamic movement of the unknown states of the drone and its moving locations. We get the advantage of Bernoulli random finite set algorithm to track the drone’s moving positions and detecting its random emission states. Hence, MIMO radar jointly makes an optimal decision on the mobility and power control to the targeted drone. Experimentation results demonstrate the performance and effectiveness of the proposed method, to intercept and destabilize the rogue drones. Additionally, by taking full consideration of the sensing and dynamic localization, we can observe and well detect the unknown emission states of the drone even when it is still moving.

Future investigations are needed for different emission signals of new drones technology, as 5G has brought brand-new cooperative environment of spectrum sensing.
